# Real-time multicompartment Hodgkin-Huxley neuron emulation on SoC FPGA

**DOI:** 10.3389/fnins.2024.1457774

**Published:** 2024-11-12

**Authors:** Romain Beaubois, Jérémy Cheslet, Yoshiho Ikeuchi, Pascal Branchereau, Timothee Levi

**Affiliations:** ^1^IMS, UMR5218, CNRS, University of Bordeaux, Talence, France; ^2^Institute of Industrial Science, The University of Tokyo, Tokyo, Japan; ^3^LIMMS, CNRS-Institute of Industrial Science, UMI 2820, The University of Tokyo, Tokyo, Japan; ^4^JSPS International Research Fellow, The University of Tokyo, Tokyo, Japan; ^5^Institute for AI and Beyond, The University of Tokyo, Tokyo, Japan; ^6^INCIA, UMR5287, CNRS, University of Bordeaux, Bordeaux, France

**Keywords:** SoC FPGA, multicompartment neurons, Hodgkin-Huxley, real-time, spiking neural network

## Abstract

Advanced computational models and simulations to unravel the complexities of brain function have known a growing interest in recent years in the field of neurosciences, driven by significant technological progress in computing platforms. Multicompartment models, which capture the detailed morphological and functional properties of neural circuits, represent a significant advancement in this area providing more biological coherence than single compartment modeling. These models serve as a cornerstone for exploring the neural basis of sensory processing, learning paradigms, adaptive behaviors, and neurological disorders. Yet, the high complexity of these models presents a challenge for their real-time implementation, which is essential for exploring alternative therapies for neurological disorders such as electroceutics that rely on biohybrid interaction. Here, we present an accessible, user-friendly, and real-time emulator for multicompartment Hodgkin-Huxley neurons on SoC FPGA. Our system enables real-time emulation of multicompartment neurons while emphasizing cost-efficiency, flexibility, and ease of use. We showcase an implementation utilizing a technology that remains underrepresented in the current literature for this specific application. We anticipate that our system will contribute to the enhancement of computation platforms by presenting an alternative architecture for multicompartment computation. Additionally, it constitutes a step toward developing neuromorphic-based neuroprostheses for bioelectrical therapeutics through an embedded real-time platform running at a similar timescale to biological networks.

## 1 Introduction

Millions of individuals globally experience the debilitating effects of neurological disorders, which significantly impact their cognitive and/or motor functions (World Health Organization, 2020). While there is a growing array of technologies and solutions being developed for the treatment of these conditions, they often only serve to slow progression or alleviate symptoms (Chin and Vora, 2014; French et al., 2016).

In addition to medical interventions involving chemical processes, artificial devices are being developed to enhance the quality of life for individuals. Bringing neuroprostheses to realization requires consideration of the behavior of biological neurons and their connections and interactions with artificial neural networks. Consequently, investigating the interaction of neuronal cell assemblies is necessary to comprehend and replicate specific behaviors driven by intrinsic spontaneous activity. Moreover, achieving long-term replacement of damaged brain regions with artificial devices necessitates an understanding of their neurophysiological behaviors.

In this context, there is a pressing need for new therapeutic approaches and technologies aimed at promoting cell survival and regenerating local circuits (Farina et al., 2021), as well as restoring long-distance communication between disconnected brain regions and circuits (Bouton et al., 2016). Therefore, the characterization and modeling of biological neural networks (Panuccio et al., 2018; Semprini et al., 2018) are crucial for the development of a new generation of neuroprostheses. These prostheses aim to mimic biological dynamics and provide adaptive stimulation at biological timescales, following the principle of electroceutics (Famm et al., 2013; Reardon, 2014; Donati and Valle, 2024).

With the advent of new neuromorphic platforms, conducting biohybrid experiments is gaining increasing relevance. This is not only crucial for advancing neuromorphic biomedical devices (Famm et al., 2013; Reardon, 2014) but also for gaining insights into the mechanisms of information processing in the nervous system. Recent advancements in neuroprostheses have significantly contributed to this progress (Panuccio et al., 2018; Semprini et al., 2018). Neuromorphic devices now have the capability to receive and process input, while also delivering their output locally or remotely through various means such as electrical, chemical, or optogenetic stimulation (Christensen et al., 2022).

The significant advancements in bioelectronics and neuroprosthetics resulted in technologies able to replace and retrain either brain (Chiappalone et al., 2022) or somatosensory functions (Raspopovic et al., 2021; Iberite et al., 2023), block seizures in epilepsy (Geller et al., 2017), and relive symptoms in neurodegenerative diseases such as Parkinson's disease (Pycroft et al., 2018; Milekovic et al., 2023).

In order to conduct bi-directional biohybrid experiments and devise bioelectrical therapeutic solutions for healthcare, such as electroceutics (Famm et al., 2013; Reardon, 2014; Donati and Valle, 2024; Di Florio et al., 2023), it is essential to incorporate real-time bio-physics interfaces and SNN processing. These components are imperative to facilitate interactions at the biological time scale (Corradi and Indiveri, 2015; Sharifshazileh et al., 2021).

A new generation of neuro/brain prostheses, termed “twins”, has emerged with the capability to replace damaged brain tissue. These innovations span from peripheral interventions (Donati and Valle, 2024; Valle et al., 2018; Romeni et al., 2020) to central nervous system interfaces (Rowald et al., 2022). Despite primarily existing as proof of concepts, neuromorphic twins hold promise for revolutionizing healthcare (Donati and Valle, 2024; Buccelli et al., 2019; Keren et al., 2019; Mosbacher et al., 2020; Beaubois et al., 2024).

Hence, this generation of neuroprostheses pushes the need for biophysically detailed neuron model. For instance, as disorders in nervous system and neuronal network can be induced from ion channel morphology (Lai and Jan, 2006; Spillane et al., 2016), the capability to reproduce the shape of the action potential with biophysical detail and biological meaningfulness to relate changes in its shape to biophysical values is important. Consequently, the most suitable candidate is the Hodgkin-Huxley (HH) paradigm (Hodgkin and Huxley, 1990) that is one of the most biologically meaningful model (Izhikevich, 2004; Brette, 2015).

While the single compartment HH model is mostly used over multicompartment model, as it is simpler and less resource-intensive, it remains limited due to its inability to capture the complex morphology of neurons (Beaubois et al., 2022). In contrast, multicompartment models offer a more comprehensive and biologically realistic approach, thus providing deeper insights into neuronal function and information processing, finding interest in multiple fields from biological interest as a study model for neurological disorders, and learning mechanism to computing interest inspired from the dendritic architecture of the neurons (Markram et al., 2015).

Multicompartmental modeling notably allows the investigation of the role of dendrites in neurons. Dendrites, particularly important regions for vital computations tied to their spatial morphology (Forrest et al., 2018), undergo learning-related changes, as evidenced in dendritic compartments (Godenzini et al., 2022). Dendrites also facilitate a greater diversity of presynaptic terminal classes, leading to different learning laws (Froemke et al., 2005; Sardi et al., 2018) and contribute to support diverse information processing strategies in neural networks (Markram et al., 2015). Moreover, they are known to exhibit physiological and morphological abnormalities during postnatal development in motor neurons affected by amyotrophic lateral sclerosis (ALS) (Martin et al., 2013) and marked changes in their structure (Fogarty et al., 2016).

Moreover, studies such as Brette (2015) shows that there are phenomena such as frequency-dependent attenuation of membrane as a function of frequency or the presence of wide variations in voltage which may be induced by the presence of active conductances distributed along the axon and dendrites. Thus, important biophysical phenomena such as spike initiation (Naundorf et al., 2006) in the axon initial segment (AIS) (Debanne et al., 2011) or in the dendrites (Gasparini et al., 2004) can be modeled. Phenomena such as dendritic spikes are, for example, known to play a part in stimulus selectivity in cortical neurons (Smith et al., 2013). Hence, the multicompartmental modeling is undoubtedly crucial to the creation of faithful and reliable model providing enough biological meaningfulness to study neurological disorders through artificial models.

The state of the art in multicompartment Hodgkin-Huxley (HH) neurons has been significantly advanced by the development and continuous updating of software platforms such as NEURON (Carnevale and Hines, 2006; Kumbhar et al., 2019; Awile et al., 2022) or integration of its computing core as in Zhang et al. (2023). NEURON is a simulation environment for modeling individual neurons and networks of neurons, allowing for the creation of complex neural models that incorporate multicompartment HH models mainly scripting in hoc with Python interfaces. While NEURON stands out for its comprehensive features and widespread use in computational neuroscience, other software platforms such as Arbor (Abi Akar et al., 2019) and Brian (Stimberg et al., 2019) also contribute to the landscape of neural simulation. Arbor is optimized for high-performance simulation of large neural networks, emphasizing multicore CPUs and GPUs, while Brian is known for its simplicity and flexibility. Additionally, an other notable GPU implementation is Kobayashi et al. (2021) that explores the use of an explicit solver to reduce computation time. Another implementation using an explicit solver designed for FPGA-based datacenter/cloud paradigm, with a focus on computational power and additional features such as gap junctions, can be found (Miedema et al., 2020; Miedema and Strydis, 2024).

Benefiting from a flexible and real-time architecture identical to BimuS (Beaubois et al., 2024), a real-time biomimetic single compartment SNN, this contribution is intended to propose a novel hardware architecture for multicompartment HH neuron emulation using SoC FPGA promoting ease of use and versatile interconnection. Furthermore, this study takes advantage of High-Level Synthesis (HLS) design methods (Cong et al., 2011; Nane et al., 2015) paired with standard hardware design to improve portability, reduce development time, and open contributions to a larger part of the community. Consequently, this system constitutes a first step toward a real-time multicompartment HH neuron emulation platform on SoC FPGA that could easily integrate biohybrid closed-loop system to explore the electroceutic approach and potentially contribute to the development of neuroprostheses and neuromorphic twins.

## 2 Materials and methods

This section introduces the system, outlining its architecture and the methods for numerical solving.

### 2.1 Neuron model

Neurons are multicompartment neurons following the Hodgkin-Huxley (HH) (Hodgkin and Huxley, 1990) paradigm that is based on the one dimensional cable equation applied to the HH paradigm corresponding to Equation 1, thus introducing the spatial dimension *x* in the equation (Carnevale and Hines, 2006).


(1) $$\begin{array}{l} \frac{1}{2\pi a}\frac{\partial }{\partial x}\left(\frac{\pi a^{2}}{R_{a}}\frac{\partial V}{\partial x}\right)=C_{m}\frac{\partial V}{\partial t}+I_{HH} \end{array}$$

where *a* is the radius of the compartment, *R*_*a*_ the resistance of the axon, *C*_*m*_ the membrane capacitance, *I*_*HH*_ the currents of the HH model, and *V* the membrane potential in the middle of the compartment.

**Figure 1:**
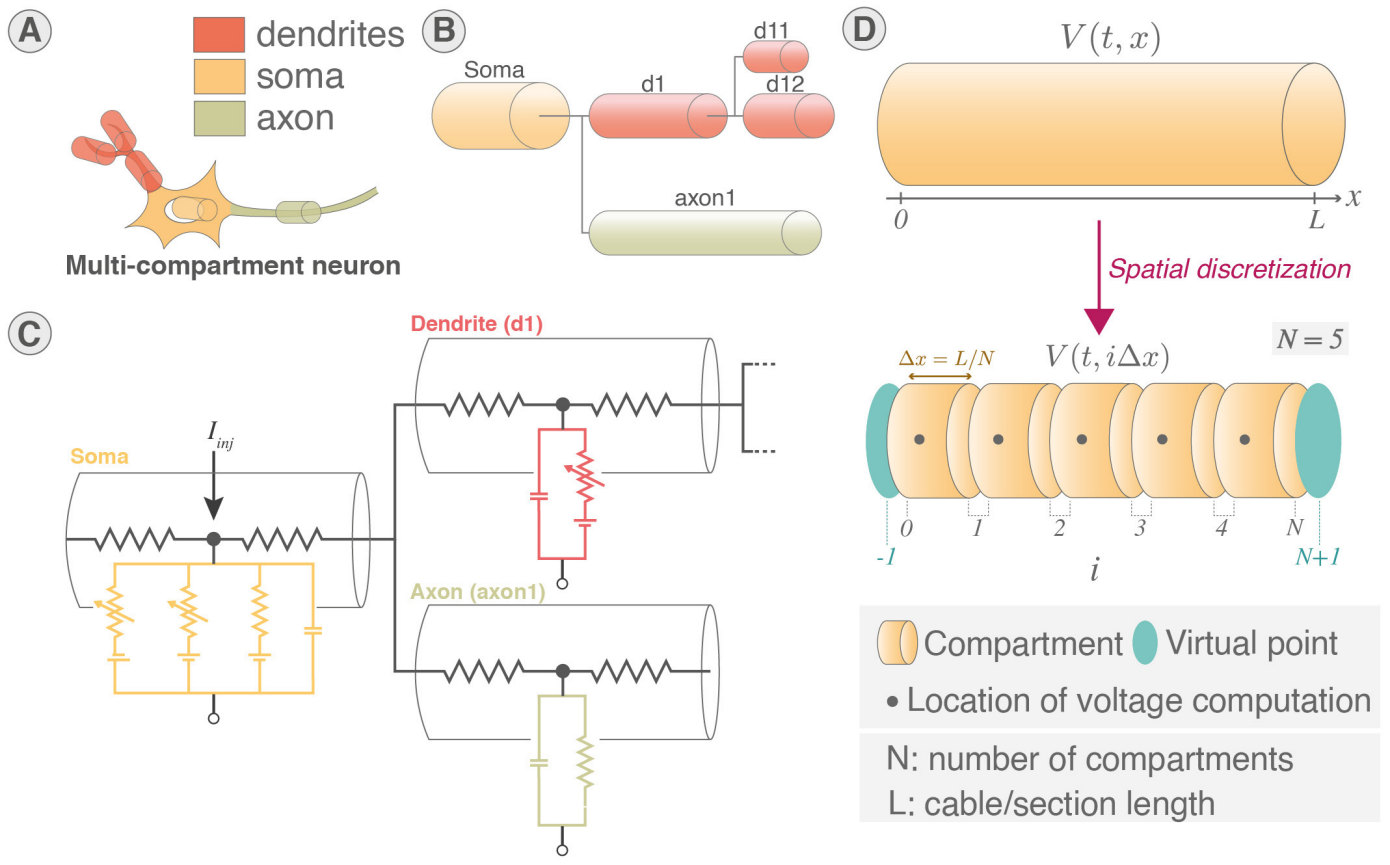
Multicompartment neuron model. **(A)** Schematic of multicompartment neuron model. **(B)** Representation of multicompartment modeling showing different parts of the neuron modeled as connected cylinders (sections). **(C)** Electrical equivalent circuit of multicompartmental neuron model. The neuron is compartmentalized in cylinder of various length and diameters representing different elements of the neuron and their properties. *I*_*inj*_ is the current injected. **(D)** Spatial discretization of a section where a cable is approximated as a series of connected cylinders named segments (or compartments). Virtual points are added at the extremities of the section to verify the no current leak condition.

Neurons implement ion channels of the Pospischil model (Pospischil et al., 2008) introducing six conductance-based currents and a stimulation current. Neurons are divided in sections that share the same electrical properties and represent different elements of the neuron similarly to Carnevale and Hines (2006) as illustrated in Figures 1A, B. An electrical equivalent circuit of the multicompartmental model using HH paradigm is shown in Figure 1C.

The spatial discretization involves the second order correct approximation of ∂^2^*V*/∂*x*^2^ (Equation 1) equated in Equation 2 (Carnevale and Hines, 2006). A representation showing cable equations discretized using “compartmentalization” that approximates the cable equations by a series of compartments (also called segments) connected by resistors is shown in Figure 1D.


(2) $$\begin{array}{l} \frac{\partial^{2}V}{\partial x^{2}}\approx \frac{V\left(x+\Delta x\right)-2V\left(x\right)+V\left(x-\Delta x\right)}{\Delta x^{2}} \end{array}$$

The discretized model can be seen as the computation of spatio-temporally continuous variables over a set of discrete points in space (“grid” of “nodes”) for a finite number of instants in time (Carnevale and Hines, 2006). Therefore, values of functions will refer at points on the grid function equated in Equation 3 (Mascagni, 1990).


(3) $$\begin{array}{l} G_{i}^{n}\equiv G\left(i\Delta x,n\Delta t\right) \end{array}$$

where Δ*t* is the time step and $\Delta x=\frac{L}{N}$ the grid width computed from *L* the length of the cable and *N* the number of spatial grid points.

The membrane potential is then evaluated at the middle of each compartment. The boundary condition that states that no axial current flows at the ends of the cable is respected by adding virtual points at the extremities of the cable.

While the use of explicit methods is suggested to be applicable for multicompartmental model solving according to Kobayashi et al. (2021), explicit methods remain limited for real-time systems because of the significant constraint imposed by the very small time step required. While the explicit Runge-Kutta-Chebyshev method with a very small time step is shown stable for multicompartment modeling (Kobayashi et al., 2021), simpler explicit solvers of lower accuracy as the Forward Euler used for the single compartment modeling are known unstable for multicompartment modeling (Carnevale and Hines, 2006).

A numerically stable solver appropriated for stiff systems and widely used is the Crank-Nicholson method. It relies on an evaluation at half a time step using Backward Euler advanced over the full interval with Forward Euler and is known stable and accurate (Carnevale and Hines, 2006; Hines, 1984). The equation applied to the membrane potential is equated in Equation 4.


(4) $$\begin{array}{l} V_{i}^{n+1}=2V_{i}^{n+\frac{1}{2}}-V_{i}^{n} \end{array}$$

The second order correct and numerically stable solution of the finite difference form of Equation 4 is expressed in Equation 5 as a tridiagonal linear system evaluated at half a time step.


(5) $$\begin{array}{l} L_{i}V_{i-1}^{n+\frac{1}{2}}+D_{i}V_{i}^{n+\frac{1}{2}}+U_{i}V_{i+1}^{n+\frac{1}{2}}=B_{i} \end{array}$$

where *L* is the lower diagonal, *D* is the main diagonal, *U* is the upper diagonal, and *B* the right-hand side of the system defined in Equation 6.


(6) $$\begin{array}{l} L_{i}=\frac{1}{2\pi a_{i}\Delta x}\frac{\pi a_{i-1}^{2}}{R_{a}\Delta x}\\ U_{i}=\frac{1}{2\pi a_{i}\Delta x}\frac{\pi a_{i+1}^{2}}{R_{a}\Delta x}\\ D_{i}=-\left(L_{i}+U_{i}+\frac{2C_{m}}{\Delta t}+g_{tot}^{n+\frac{1}{2}}\right)\\ B_{i}=\frac{2C_{m}}{\Delta t}V_{i}^{n}+g_{Na}^{n+\frac{1}{2}}E_{Na}+g_{Kd}^{n+\frac{1}{2}}E_{K}+g_{M}^{n+\frac{1}{2}}E_{K}\\ +g_{T}^{n+\frac{1}{2}}E_{Ca}+g_{L}^{n+\frac{1}{2}}E_{Ca}+g_{Leak}^{n+\frac{1}{2}}E_{Leak}\\ \;+g_{Syn}^{n+\frac{1}{2}}E_{syn}+\delta_{i0}\frac{I_{inj}(t)}{2\pi a\Delta x} \end{array}$$

with *g*_*Na*_, *g*_*Kd*_, *g*_*M*_, *g*_*T*_, *g*_*L*_, *g*_*Leak*_, and *g*_*Syn*_ representing the conductances for sodium, potassium, slow voltage-dependent potassium, high-threshold calcium, low-threshold calcium, leakage currents, and receptor-dependent synaptic conductance, respectively. *g*_*tot*_ the sum of all the conductances. *E*_*Na*_, *E*_*K*_, *E*_*Ca*_, *E*_*Leak*_, and *E*_*Syn*_ the reversal potentials, respectively, for sodium ions, potassium ions, calcium ions, leakage, and receptor-dependent synaptic currents. *I*_*inj*_ the current injected, and δ_*i*0_ the Kronecker delta.

**Figure 2:**
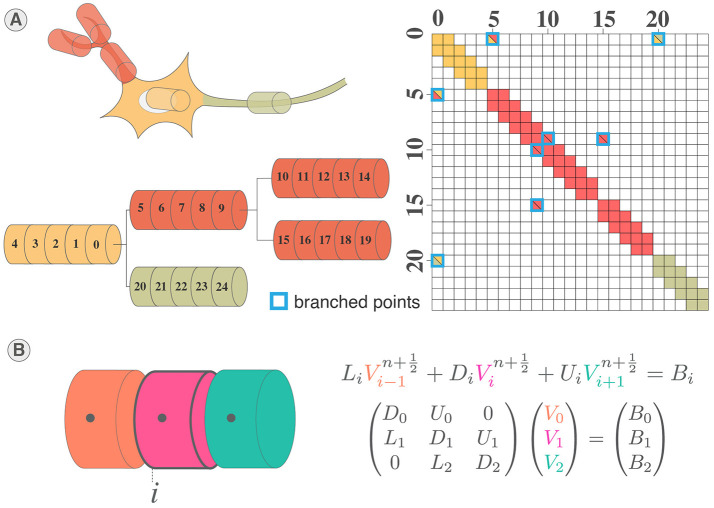
System of equations and solving of multicompartment model. **(A)** Illustration of mainly tridiagonal matrix with sparse coefficients (Hines matrix) at branches points generated by the multicompartmental neuron structure. **(B)** Illustration of the tridiagonal systems of equations corresponding to the computation of the membrane potential in a section of a multicompartmental neuron.

The complete structure of the neuron corresponds to a tree of unbranched cables (sections) divided in N segments (or compartments), thus adding off-diagonal coefficients to the tridiagonal linear system (Hines, 1984) (Figures 2A, B). Through wise numbering of the nodes in the tree, the tridiagonal matrix resulting is solvable thanks to Hines matrix solver.

All the segments of neurons can be connected through fully configurable biomimetic synapses mimicking AMPA, NMDA, GABA_*A*_, and GABA_*B*_ synaptic receptors (Destexhe et al., 1998) to allow fast and slow synaptic excitation or inhibition.

### 2.2 Computation core

**Figure 3:**
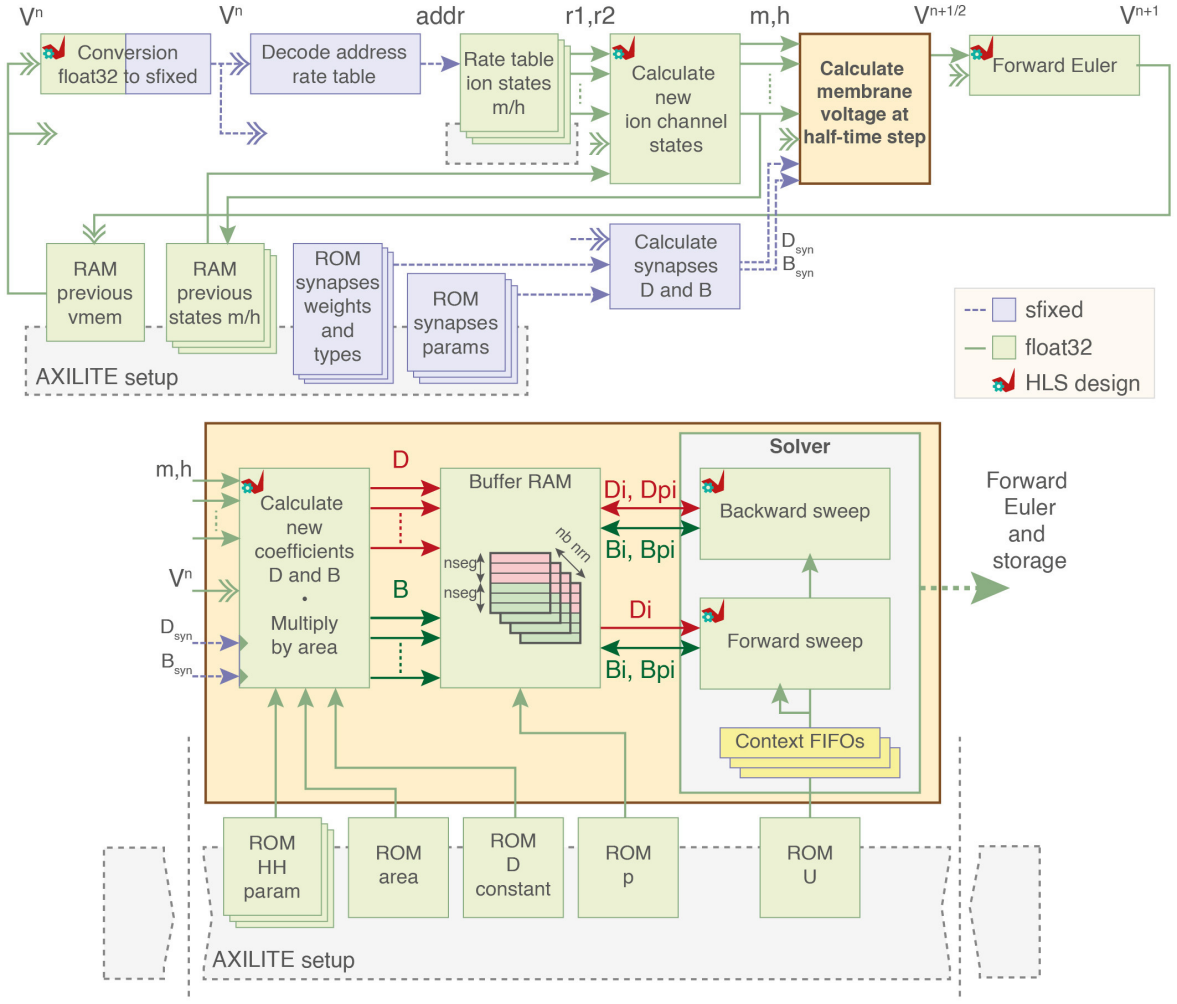
Block diagram of the computation core. Ion channel states variables are calculated from premultiplied rates and used to compute ion currents as two coefficients D and B. Dual-port buffer RAMs for D and B of each neuron load and store data to and from the forward and backward sweep cells. Parameters of the model are stored in block RAMs initialized by the PS through AXI-Lite. sfixed; signed fixed-point. float32; 32-bit floating-point. HLS, High-Level Synthesis using AMD Vitis HLS.

The architecture of the pipelined computation core using 32-bit floating-point coding is presented in Figure 3.

The computation core employs the Crank-Nicholson solver for its numerical stability and accuracy (Carnevale and Hines, 2006; Beaubois et al., 2022). Instead of relying on resource-intensive matrix inversion, a more efficient alternative is employed utilizing strategic compartment numbering and the Hines algorithm (Hines, 1984). Originally designed for CPU architecture, a variant of this algorithm utilized in the GPU-oriented simulator Arbor developed by the Human Brain Project community (Abi Akar et al., 2019) and in Valero-Lara et al. (2018) was implemented.

The algorithm uses a parent node vector *p* so the matrix can be stored using two vectors corresponding to the main diagonal *D* and upper diagonal *U*. Branching points are then reconstructed due to the parent node vector. Algorithm 1 solves the matrix, and Algorithm 2 generates the main diagonal *D*.

**Algorithm 1:**
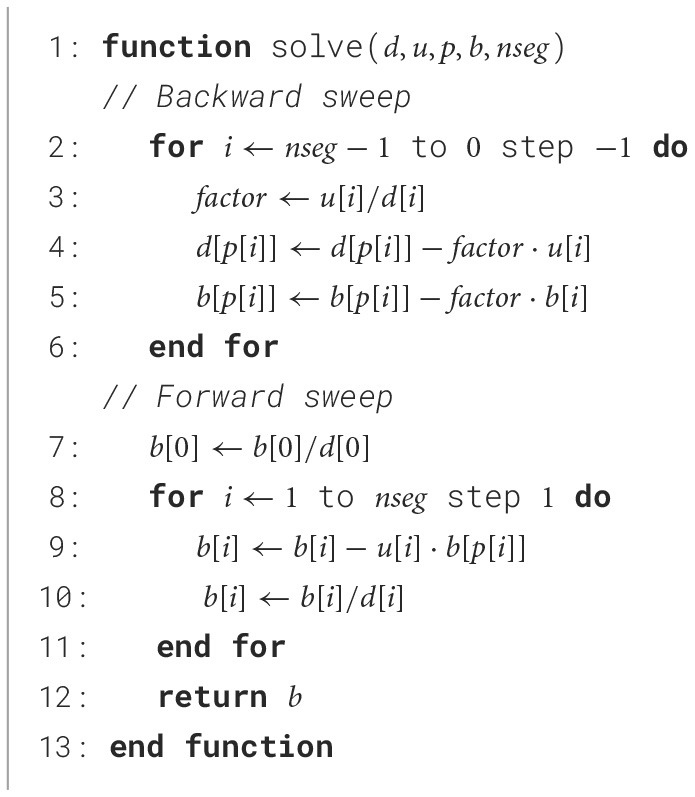
Hines algorithm used in Abi Akar et al. (2019).

**Algorithm 2:**
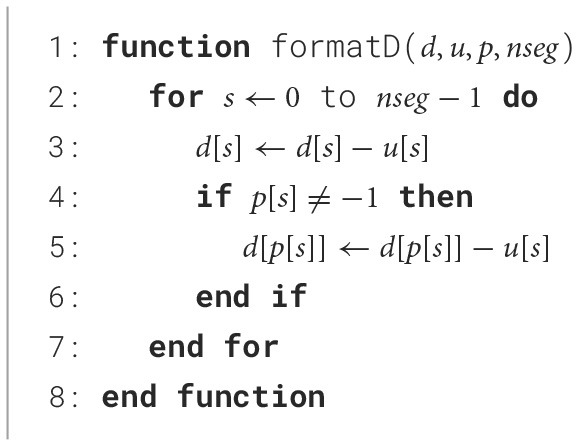
Initialize format main diagonal.

The computation of ion channel states is based on “premultiplied” HH rate function tables as described in Hines (1984), simplifying computation to a single multiply and add from table values looked-up based on the membrane voltage (Equation 7). This method eliminates the FPGA-specific limitations for complex mathematical functions such as division and exponential.


(7) $$\begin{array}{l} x_{n+1}=r_{1}\left(V_{n}\right)\times x_{n}+r_{2}\left(V_{n}\right) \end{array}$$

where *x*_*n*+1_ and *x*_*n*_ are, respectively, the new and current value of the ion channel state, *V*_*n*_ is the membrane voltage at previous time step, and *r*_1_ and *r*_2_ are the ion rate tables decoded from the membrane voltage.

The premultiplied tables for common equations of ion channel states correspond to Equations 8, 9.


(8) $$\begin{array}{l} r_{1}\left(V\right)=1-dt\times \left(\alpha_{x}\left(V\right)+\beta_{x}\left(V\right)\right)\\ r_{2}\left(V\right)=dt\times \alpha_{x}\left(V\right) \end{array}$$

(9) $$\begin{array}{l} r_{1}\left(V\right)=1-\frac{dt}{tau_{x}\left(V\right)}\\ r_{2}\left(V\right)=\frac{dt\times x_{\infty }\left(V\right)}{tau_{x}\left(V\right)} \end{array}$$

where *r*_1_ and *r*_2_ are the pre-computed rate tables for ion channel states decoded from the membrane voltage, *dt* the time step in ms, and *tau*_*x*_, *x*_∞_, α_*x*_, and β_*x*_ the equations of the ion channel state depending on the formalism used.

The calculation module for synapses is adapted from BiœmuS (Beaubois et al., 2024) to match multicompartment equations allowing a fully configurable synaptic connection so all nodes can be connected and independently weighted. As for BiœmuS computed using 18-bit fixed-point coding, parameters of the synaptic models are set through AXI-Lite and pre-computed tables are used to encode the exponential rates of the synaptic receptors.

As the solving algorithm of the matrix includes sequential divisions and multiplications, the stability of the solving requires high accuracy that is better translated by floating point. Indeed, the coefficients greatly vary with the geometrical dimensions of the neurons that may create larger orders of magnitude that are delicate to handle with fixed point. Hence, 32-bit floating point was implemented to offer floating-point accuracy with limited resource consumption compared to 64-bit floating-point coding that shows significantly higher implementation cost in programmable logic for a limited gain in accuracy for this application.

The parameters of the model are loaded in BRAM through AXI-LITE registers controlled by the software application, hence facilitating interconnect of several cores thanks to AXI (Advanced eXtensible Interface) protocol. The AXI communications are clocked at 100 and 200 MHz, respectively, for ZynqMP and Versal architecture, while the rest of the design operates at 200 and 400 MHz, respectively, for ZynqMP and Versal architecture expect for the synapses that are clocked at 400 MHz for both architectures.

The computation core starts by loading the previous membrane voltage to decode the rate tables for ion channel states computation and allow computation in other modules. The computation of the values for ion currents is output as two separate coefficients *D* and *B*. The system solving includes two computation blocks that perform the backward and forward sweep of the Algorithm 1 along with context FIFOs to keep track on the solving state.

The coefficients for each segment are then stored in one dual-port RAM (lower addresses is *D* and upper addresses are *B*) with one BRAM per neuron. These BRAMs act as buffer memory for the operations of the forward and backward sweep by loading and storing the values of segments at each iteration until the matrix is completely solved.

Main computation modules were designed using High-Level Synthesis (HLS) through AMD Vitis HLS facilitating optimization, portability, and integration. Optimal HLS modules can be generated for each target and integrated easily in the design by adjusting the latency in the generic HDL.

A comparison with the NEURON software (Carnevale and Hines, 2006) has been conducted in prior work of the team (Beaubois et al., 2022) for the soma of a motor neuron including only sodium, potassium, and leak currents modeled using five segments of identical length, diameter, and properties. The emulation shows a slight difference with the NEURON software explained by the difference of solver that is CVODE for NEURON and Crank-Nicholson for the software emulation but mostly by the hardware architecture constraints in terms of data coding and operations. Indeed, the hardware is operating on 32-bit floating point by a FPGA instead of 64-bit floating point on software with a CPU for NEURON.

The selected architecture for the computation core designed is promoting the scaling in segments rather than neurons as the emphasized is put on the morphology of neurons better translated by a high number of segments. For example, the allocation of one BRAM per neuron allows for the storage of up to 576 segments per neuron.

### 2.3 Platform

The system corresponds to the integration of the computation core on SoC FPGA, specifically AMD Zynq UltraScale+ MPSoC and AMD Versal Adaptive SoCs, that can be organized in two parts: Programmable Logic (PL, i.e., FPGA) and processors in a Processing System (PS) part. The implementation on the low-cost System-on-Module (SOM) K26 (ZynqMPSoC architecture) embedded on either AMD Kria KR260 Robotics Starter Kit or Kria KV260 Robotics Starter Kit is capable of running up to 16 neurons of 64 compartments each with up to 1,048,576 fully configurable continuous conductance-based synapses. It includes on-board monitoring and offers external communication options such as Ethernet and expansion PMODs (standard peripheral module interface) allowing different compromises for monitoring and interconnection. Implementation on more performing architecture, such as AMD Versal Premium Series (VPK120 Evaluation Kit), increases the number of compartments to 96 segments each for an identical number of neurons using the same computation core.

**Figure 4:**
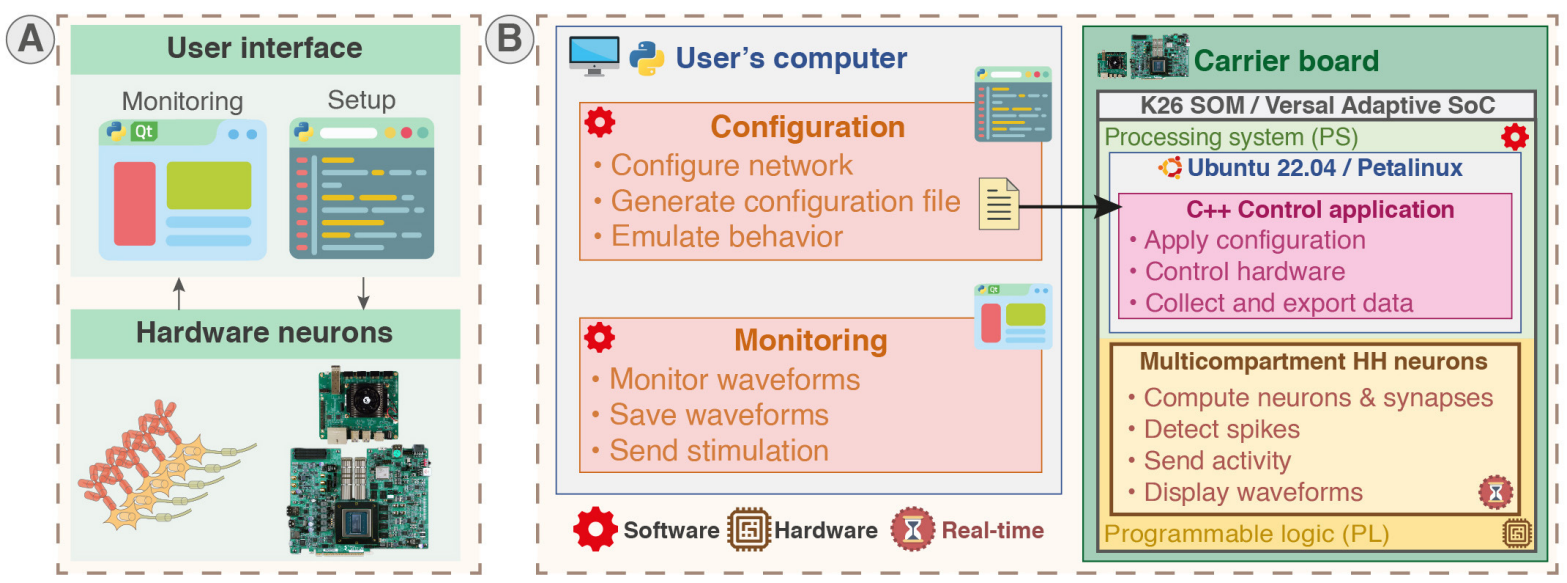
System overview of the real-time hardware-based emulator for multicompartment Hodgkin-Huxley neurons. The nature of each part of the system (software or hardware design) is identified by red and brown symbols. The on-board configuration and monitoring are also available but not displayed on the figure. **(A)** Overview of the platform integrating hardware neurons allowing users to configure and monitor the system through Python scripts and Qt-based GUI. The platform allows for real-time emulation of multicompartment Hodgkin-Huxley (HH) neurons with configurable parameters. **(B)** The system can run on carrier boards integrating different architecture of SoC featuring CPU in a processing system part (PS) and FPGA in a programmable logic part (PL), being either Zynq MPSoC through the System-on-Module K26 (SOM) or AMD Versal Adaptive SoC. The real-time hardware neurons are implemented in PL part and controlled through a C++ control application running in the PS part. The PS part runs either a Canonical Ubuntu or a custom Linux (generated using PetaLinux toolchain) allowing standard interfacing and operation. Monitoring is performed by a Qt-based GUI and setup by configuration scripts in Python ran either on-board or on another computer.

The platform, allowing for emulation and monitoring as presented in Figure 4A, was developed using three different languages that correspond to three distinct parts as shown in Figure 4B.

**Figure 5:**
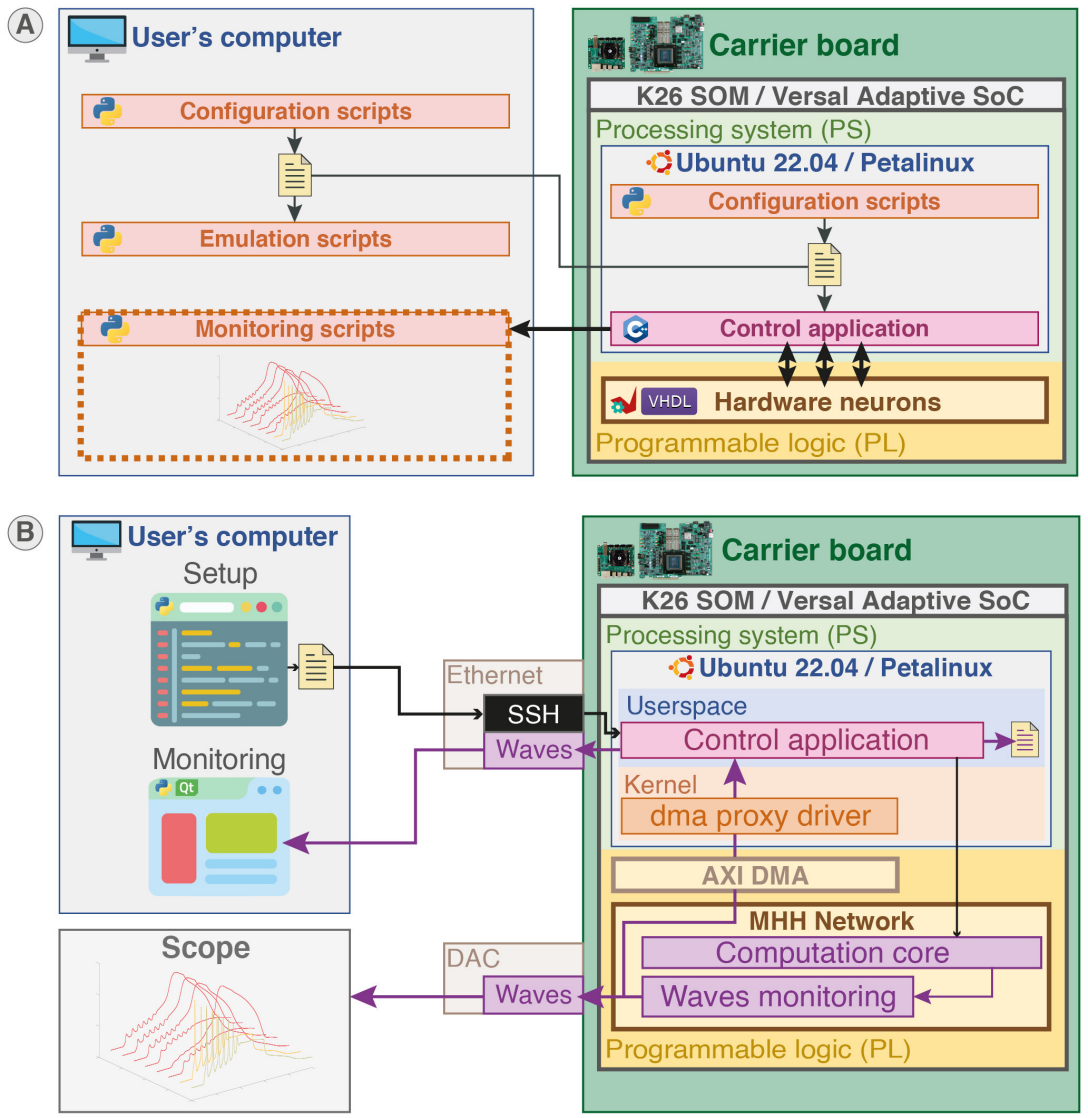
Platform configuration, control, and monitoring. **(A)** Platform configuration. Configuration scripts (Python) ran either locally or on another station generate configuration files. The configuration files are loaded by the control application (C++) running in the user space of the operating system (Canonical Ubuntu or custom Linux) in the PS part to set up the SNN in PL part. Emulation scripts allow emulation of the configuration beforehand to predict the behavior. **(B)** Platform control and monitoring. The platform can be controlled and monitored either remotely via SSH or on-board from the desktop through the control application. The membrane potentials of neurons can be monitored concurrently using Ethernet forwarding, on-board file saving, and visualization on scope by probing the Digital-to-Analog Converter (DAC).

Python language is used for the configuration scripts and monitoring to provide user-friendly and rapid-prototyping as it is aimed to be used by non-specialists. The C++ language is used to develop the application responsible for setup and control in the PS part to provide better performances and proximity with hardware. VHDL was used to describe the hardware circuit in the PL part that implements the computation core of the neural network. Additionally, C++ code was used to generate the HLS IP used in the computation core. Figure 4B illustrates the different parts of the system and indicates their hardware or software nature for a configuration and monitoring on an external computer. The configuration of the network in Python can also be executed locally due to the operating system running in PS as shown in Figure 5A.

All parameters of the HH model including the ion channels equations and geometrical properties are configurable from configuration scripts, enabling the emulation of various neuron types and morphologies, such as dendritic trees. Similarly, all parameters of the synapses are configurable via the configuration scripts, allowing for the emulation of various network topologies with great detail, due to fully configurable synapses that can connect all nodes. The platform is capable of running either a commercial Linux operating system with Canonical Ubuntu to maximize compatibility and stability or custom Linux generated using AMD PetaLinux toolchain which allows easier customization such as pre-empted kernel.

The system allows for different monitoring channels illustrated in Figure 5B. The membrane potential waveforms of up to 64 compartments can be retrieved in the user space of the operating system from the computation core due to a DMA (Direct Memory Access) interfaced with AXI and controlled by a kernel driver. Then, the waveforms can be either stored locally or forwarded through Ethernet using the open-source networking library ZeroMQ. Another monitoring channel directly connecting to the hardware is available via a DAC (Digital-to-Analog Converter) outputting up to eight membrane voltages waveforms.

The hardware computation core computing the neurons as well as DAC monitoring is running in hard real time at a period of 31.25 μs, while the software monitoring through DMA is running in soft real time, implying fluctuating latency potentially larger than the deadline that is, however, not inducing a failure of the system, at a programmable period between 1 and 15 ms. Indeed, the hard real-time operation of the computation core is ensured by its implementation in programmable logic.

## 3 Results

### 3.1 Performances

The implementation of a single computation core was performed on AMD Zynq UltraScale+ MPSoC architecture (ZynqMP) on AMD Xilinx KR260 Robotic Starter Kit (KR260) and AMD Versal Adaptive SoCs (Versal) on Versal Premium Series VPK120 Evaluation Kit (VPK120). The computation core is capable of emulating in real-time 16 neurons of 64 segments each on KR260 and 16 neurons of 96 segments each on VPK120. This capability enables the exploration of parameter sets, particularly for affected neuron models, by emulating multiple neurons simultaneously in parallel. Through hardware generics, the computation core can be adjusted to compromise between the number of neurons and the number of segments. For instance, reducing the number of neurons would permit the implementation of more segments. The evaluation of the maximum number of segments that could be implemented for a computation core of a unique neuron is equated in Equation 10 (100 segments for KR260 and 150 for VPK120).


(10) $$\begin{array}{l} nbsegments_{max}<\frac{dt\times f_{clk}}{lat_{load\;context}+lat_{backward\;sweep}+lat_{forward\;sweep}} \end{array}$$

where *nbsegments*_*max*_ is the maximum number of segments that can be implemented for one neuron, *dt* the time step, *f*_*clk*_ the clock frequency, *lat*_*loadcontext*_ the latency in clock cycles to load the solver context, and *lat*_*backwardsweep*_ and *lat*_*forwardsweep*_ the latencies in clock cycles to compute one operation of the backward and forward sweep.

**Figure 6:**
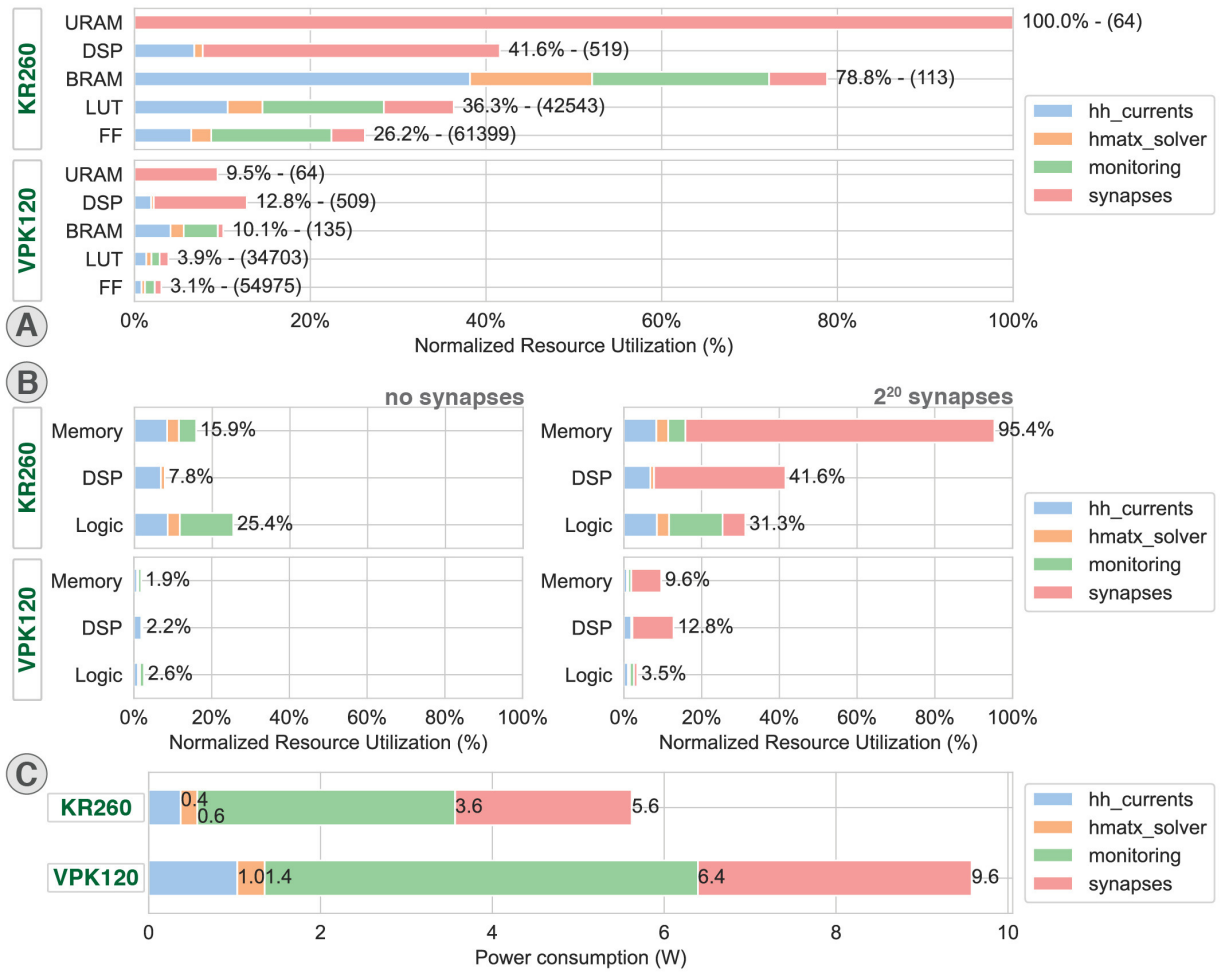
Resource utilization and power consumption. Distributed resource utilization and power consumption on AMD Xilinx KR260 Robotic Starter (KR260) and Versal Premium Series VPK120 Evaluation Kit (VPK120) for one computation core. hh_currents: computation of the ion currents of the HH model and parameters storage. hmatx_solver: Hines matrix solver computation paired with the context and buffer memory. monitoring: DMAs, related buffer memories and CPUs. **(A)** Detailed resource utilization exported from Vivado 2023.2. **(B)** Resource utilization represented by main resource groups exported from Vivado 2023.2. **(C)** Distributed power consumption exported from Vivado 2023.2.

The resource utilization reports are compared in Figures 6A, B, respectively, showing the detailed utilization in components and summarized as memory, DSP (Digital Signal Processing), and logic for the main modules of the design. While the implementation on KR260 shows an overall moderate usage of the resources, except for the BRAM, the implementation on VPK120 shows significantly lower utilization percentage. This difference is explained by a larger availability of resources but also a reduced utilization of certain components due to improved components such as native floating-point DSP or new network of interconnect using Network-on-Chip (NoC). The high BRAM usage is due to the large number of parameters that need to be stored for the multicompartment HH model, as well as the large FIFO operating in packet mode for monitoring.

The 32-bit floating-point computation of the HH currents shows an overall low utilization of DSP, LUT (LookUp Table), and FF (Flip-Flop) for KR260 due to operations being mostly multiplications that translate to an even lower utilization on VPK120 that benefits from a more recent architecture integrating native floating-point DSP. The Versal architecture also leverages the use of Network-on-chip (NoC) to reduce FF and LUT used for AXI interconnect.

Because of the use of a pipeline solver cell, the resource utilization associated with the matrix solver is significantly lower than the other modules. Hence, adding parallel solving cells would allow a consistent improvement of performances for an acceptable cost in resource utilization.

The system also benefits from a low power consumption of 5.6 W on KR260 and 9.6 W on VPK120 (Figure 6C) compared to CPU and GPU solutions usually larger than the tens of watts. The main power consumption corresponding to the monitoring was associated with AXI DMA and processors.

Since real-time multicompartment Hodgkin-Huxley (MHH) emulation is quite rare in the literature, this study is compared to other embedded implementations of biomimetic neural networks aimed at biohybrid interaction, as shown in Table 1, without limiting the comparison to multicompartment models. While prominent neuromorphic platforms typically focus on neuromorphic computing and are usually not suited for biohybrid interaction, low-power Loihi chips (Davies et al., 2018; Orchard et al., 2021) and the demonstrated mobile setup of BrainScaleS-2 (Pehle et al., 2022; Stradmann et al., 2022) computing faster than real-time show sufficient suitability with embedded applications to be included.

**Table 1:** Comparison with embedded systems implementing biomimetic neural network for biohybrid interaction and mobile solutions of prominent neuromorphic platforms [Loihi neuromorphic chips (Davies et al., 2018; Orchard et al., 2021) and mobile demonstration of BrainScaleS-2 (Pehle et al., 2022; Stradmann et al., 2022)].

	**Hardware**	**Neuron model**	**Synapse type**	**# neurons**	**# synapses**
This work	SoC FPGA	**MHH**	Continuous	16	1,048,576
BioemuS (Beaubois et al., 2024)	SoC FPGA	HH	Continuous	1,024	1,048,576
Xu et al. (2018)	FPGA	HH	Continuous	~10,000	~3,200
Cheslet et al. (2024)	FPGA	HH	Continuous	100	100
Buccelli et al. (2019)	FPGA	IZ	Continuous	100	2,500
Keren et al. (2019)	VLSI/FPGA	LIAF	Continuous	2,880	12,700,000
Vallejo-Mancero et al. (2024)	SoC FPGA	QLIF	Continuous	1,280	40,960
Hwang et al. (2023)	SoC FPGA	LIF	Event driven	60	3,600
Stradmann et al. (2022)	ASIC/SoC FPGA	AdEX	Event driven	512	131,072
Loihi (Davies et al., 2018)	ASIC	LIF	Event driven	128,000	128,000,000
Loihi 2 (Orchard et al., 2021)	ASIC	LIF, AdEX, IZ	Event driven	1,000,000	120,000,000
	**# segments**	**Real-time**	**Application focus**	**Interface**	
This work	**64/neuron**	1 ×	Biohybrid interaction	Ethernet, DAC, GPIO	
BioemuS (Beaubois et al., 2024)	/	1 ×	Biohybrid interaction	Ethernet, Wi-Fi, DAC, GPIO	
Xu et al. (2018)	/	1 ×	Biohybrid interaction	Ethernet, GPIO	
Cheslet et al. (2024)	/	1 ×	Biohybrid interaction	Wi-Fi, GPIO	
Buccelli et al. (2019)	/	1 ×	Biohybrid interaction	UART	
Keren et al. (2019)	/	1 ×	Biohybrid interaction	Ethernet	
Vallejo-Mancero et al. (2024)	/	1 ×	Biohybrid interaction	Ethernet, HDMI	
Hwang et al. (2023)	/	1 ×	Biohybrid interaction	GPIO	
Stradmann et al. (2022)	Emulable	1,000 ×	Neuromorphic computing	USB, SDXC, I2C	
Loihi (Davies et al., 2018)	/	>1 ×	Neuromorphic computing	Proprietary asynchronous	
Loihi 2 (Orchard et al., 2021)	/	5,000 ×	Neuromorphic computing	SPI, GPIO, Ethernet	

Bold values emphasize points of novelty for comparison presented in this work.

This iteration on SoC FPGA shows promising results supported by the capability of emulating up to 16 neurons of 64 segments each parallelly in real-time per computation core where current solutions are showing much higher computation times. As for example, the software emulation of 1 s of a similar structure using NEURON took an average 3.5 s for 1 neuron on an Intel i7-10875H (without synapses).

Another benefit from the system is its versatile communication as it shares the same system integration as BiœmuS (Beaubois et al., 2024) that shows various communication interfaces and their potential for biohybrid experiment setups. As for example with the current solution, the waveform monitoring using the DAC allows monitoring up to eight membrane potentials in real-time per DAC while the transfer to PS allows monitoring of up to 64 membrane potentials that can be forwarded through Ethernet. Furthermore, considering the affordable price of the KR260 board along with the performances obtained for this entry-level FPGA, the system benefits from a great affordability.

### 3.2 Application

To offer a tangible demonstration case of the system, an application targeting a model of neurons was affected by ALS, a rapidly progressive and devastating neurodegenerative disease that targets principally motor neurons.

Motor neurons affected by ALS, specifically motor neurons of SOD1 mice at embryonic state, were modeled from patch clamp recordings (Branchereau et al., 2016, 2019; Martin et al., 2020). The models were developed with a high level of biological meaningfulness. The dynamics of the neurons were reproduced accurately because of a modeling based on patch clamp recording of each ion channels. Most notably, the models include highly detailed modeling of the morphology of the neuron, thus enforcing the use of a multicompartment modeling.

The models were developed using the NEURON software, thus not adequate for real-time emulation of a network, hence encouraging the development of this system to emulate multicompartmental neurons.

**Figure 7:**
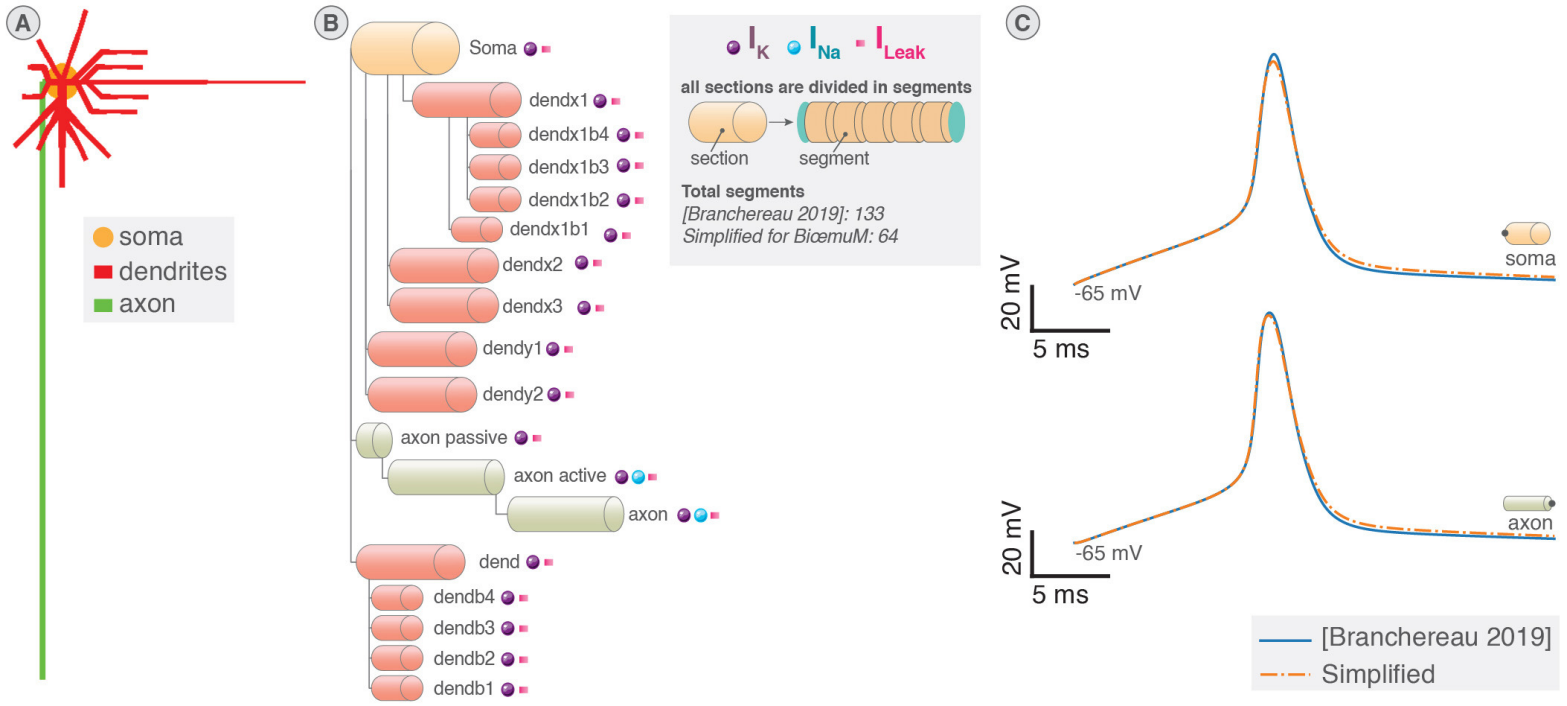
Demonstration case with a model of motor neuron. The multicompartment model of motor neuron at day E13 from Branchereau et al. (2019) designed using NEURON software has been reduced to 64 segments to allow emulation by the system. **(A)** Morphology schematic of the motor neuron at day E13. **(B)** Morphology of the neuron decomposed in sections of varying geometrical and electrical properties (length, diameter, ion, and leakage currents). Sections are decomposed in fewer segments (or compartments) in the simplified modeling. **(C)** Comparison of the evolution of the membrane potential in response to a 15 ms stimulation pulse inserted in the soma. Membrane potentials are recorded in the soma and at the end of the axon.

The model used for this application is the motor neuron at day E13 presented in Branchereau et al. (2016) that implements 133 segments (or compartments) distributed in soma, axons, and dendrites sections. The morphology of the neuron generated from the NEURON model is presented in Figure 7A. The currents involved in the model are the potassium, sodium, and leakage currents that show different conductances depending on the section. As for instance, only the active axon and the rest of the axon integrate sodium current. Figure 7B recapitulates the morphology of the neuron and shows how the sections are connected.

The model was reduced to a total of 64 segments to implement 16 neurons to match the computation core capabilities while preserving the sections and their interconnections. The simplified model was compared to the original model in NEURON software in response to a stimulation of 15 ms inserted in the soma to assess the coherence of the simplified model as shown in Figure 7C. While the simplified model is not capable of accurately reproducing the spatial morphology of the neuron, its accuracy remains satisfying in this application aiming to validate the system and showcase its potential. Indeed, the membrane potential of the simplified model is closed to the original at two distant points being the soma, where the stimulation is inserted, and at the end of the axon.

The parameters of the simplified model were then translated in the configuration scripts that allow to generate the configuration file and emulate its behavior in software. The configuration file generated was then ran on the KR260 board, and the membrane potentials of the 64 segments were monitored using the local file saving through DMA.

**Figure 8:**
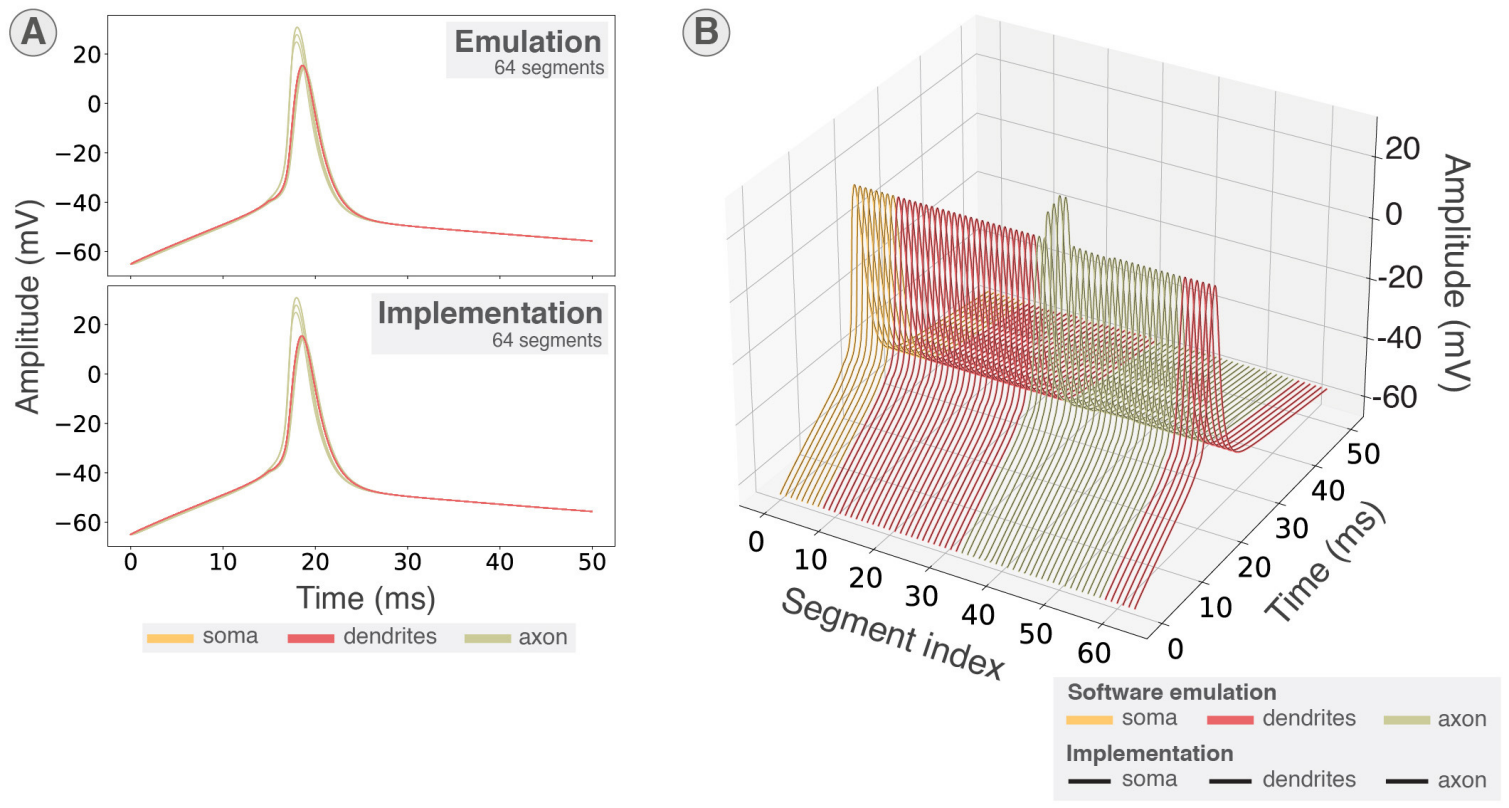
Validating hardware implementation. Comparison of membrane potentials in software emulation through the Python scripts and hardware implementation. Membrane potentials in implementation were recorded using the on-board file saving through DMA. **(A)** All 64 segments overlapped in both emulation using the Python scripts and implementation on KR260. **(B)** All 64 segments sorted by segment index for both emulations using the Python scripts and implementation on KR260. Membrane potentials retrieved from the hardware emulation are shown in black for all segments.

The membrane potentials obtained were then compared to the software emulation as shown in Figures 8A, B. Figure 8A presents the 64 segments of a neuron overlapped, showing that the action potentials in all compartments highlight the correspondence between software and hardware emulation. Figure 8B shows the membrane potentials arranged by segment index for both software emulation and hardware emulation, allowing visualization of all membrane voltages and the fitting of the hardware emulation with the reference. Hence, these results validate the implementation of the system and demonstrate its ability to emulate multicompartmental neurons in real time.

Another demonstration case highlights how tuning system parameters can adapt to various neuron models and network topologies. This case demonstrates various synaptic receptor types by integrating a neuron model with different ion channel equations and parameters, specifically using the Fast Spiking (FS) neuron model from the Pospischil model (Pospischil et al., 2008).

**Figure 9:**
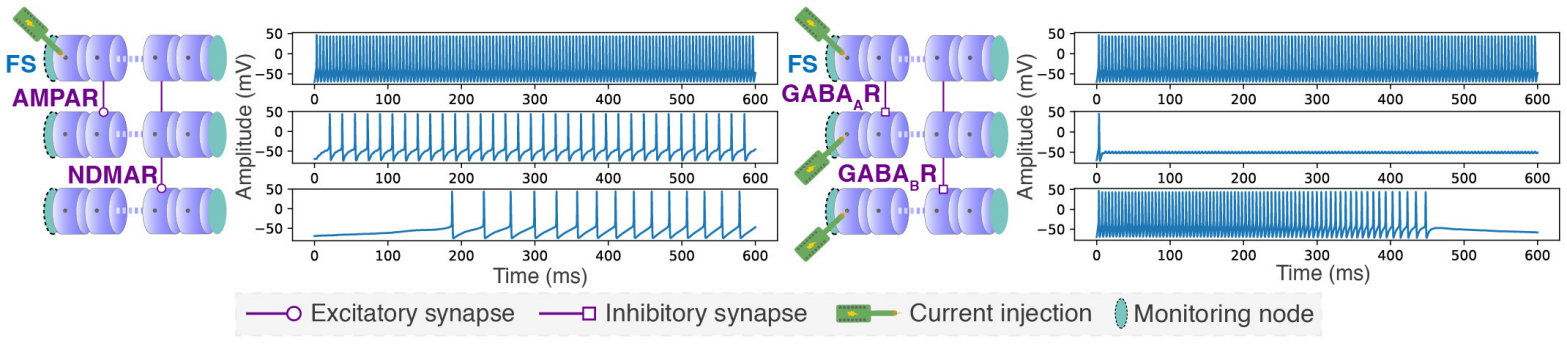
Showcasing synaptic connection types and a different neuron model. Slow and fast excitation and inhibition modeled using a conductance-based synaptic receptor model with parameters from Destexhe et al. (1998) on Fast Spiking (FS) neurons with parameters from Pospischil et al. (2008). The synaptic receptor conductances were set to *g*_*AMPAR*_ = 0.0875 *nS*, *g*_*NMDAR*_ = 0.45 *nS*, *g*_*GAB*_*A*__*A*_*R*_ = 0.15 *nS*, and *g*_*GAB*_*A*__*B*_*R*_ = 1.5 *nS*. Additionally, synaptic weights were multiplied by 128. Injected stimulation current was set to 0.3 *nA*.

Fast Spiking (FS) neuron somas, with a length of 70 μm and a diameter of 9 μm, were modeled using 64 compartments, involving the implementation of different parameters and ion channel equations compared to those used for the previously presented motor neuron. AMPA and NMDA receptors, which mediate fast and slow excitations, respectively, were demonstrated by creating synapses from a stimulated neuron to a non-stimulated neuron to observe excitatory activity. Similarly, GABA_*A*_ and GABA_*B*_ receptors, responsible for fast and slow inhibition respectively, were demonstrated by creating synapses between stimulated neurons to observe inhibitory activity. The configurations were emulated in hardware using six neurons with increased synaptic weights to quickly observe the effects of synaptic receptors through a single synapse. This demonstration case is illustrated in Figure 9.

## 4 Discussion

The system could find several application for the study of neurological disorders. As for instance, this system could serve as a highly biomimetic stimulation source in a neuromorphic-based open-loop setup for neuroprosthetic applications to be exploited in post-stroke rehabilitation studies (Panuccio et al., 2018; Semprini et al., 2018; Di Florio et al., 2023). Additionally, the system could serve as a tool for studying the biohybrid interactions crucial for advancing neuroprostheses, akin to Beaubois et al. (2024). As the system shares the same design base as Beaubois et al. (2024), additional features such as Wi-Fi monitoring could also be added seamlessly to the system on the same principle.

The main limitation of the current solution limiting its potential is the number of neurons and segments. This could be greatly improved by alleviating the current bottleneck created by the usage of only one pipelined solver cell by implementing multiple parallel backward and forward cells. Considering the resource footprint of the backward and forward cells, multiple cells could be implemented in one core, allowing for more neurons to be computed in the same time. Additionally, for larger targets such as Versal Adaptive SoCs, performances could be improved because of the larger amount of resources available allowing for the implementation of multiple cores as well as greater optimizations in terms of computation architecture through native floating-point DSP and higher clocking frequency.

Concerning the scaling of the system, as the computation core is interfaced using AXI protocol, interconnection of modules is simplified and facilitates the implementation of multiple cores. Hence, this design enables significant scalability on larger targets by integrating additional cores. Furthermore, because of the various communication interfaces proposed by the SOM K26 carrier boards, such as Ethernet, the system could support clusters of targets, each with multiple cores, allowing for a larger number of neurons or segments. Additionally, since the hardware computation core features are adjustable from HDL generics, accommodating different configurations to optimize either the number of neurons or segments, to adapt to the specific needs of real-life neuronal networks, may benefit from various topologies either favoring the number of segments or neurons. Along with the tuning of network topologies, the acceptable level of accuracy of the model compared to other software emulation can be compared with the software model running in Python to allow users to evaluate their accuracy criteria based on their specific needs. Along with tuning network topologies, the accuracy of the model can be compared to other software emulations through the software emulation in Python, allowing users to evaluate accuracy based on their specific needs.

A promising enhancement to the computation core could involve leveraging the Artificial Intelligence Engines (AIE) integrated into AMD Versal AI Core and AI Edge series. This could lead to more efficient system solving or easier implementation of alternative algorithms, capitalizing on the GPU-like architecture of the cores. Similarly to the HLS-generated computation modules, AIE-based computation modules could be linked with the generic HDL.

## 5 Conclusion and perspectives

This study explores an alternative platform for multicompartment HH neuron emulation utilizing an architecture underrepresented in literature for this specific application. It leverages SoC FPGA architecture that combines programmable logic and processors to integrate real-time computation with FPGA capabilities, while also providing a standardized interface through the CPUs and operating system at low latency.

This study highlights the potential of the platform to enable real-time emulation while enhancing accessibility, portability, and interconnection capabilities.

Indeed, real-time emulation capability, that is a crucial requirement for the realization of electroceutic therapies, paves the way for this system to be used as a novel tool to drive stimulation at a higher level of biological meaningfulness through the use of multicompartmental model.

## Data Availability

The code related to this work is available at https://github.com/Ceramic-Blue-Tim/bioemum. The code and data are available from the corresponding authors upon reasonable request.
